# On the Treatment of Abscess in the Liver

**Published:** 1829-04-01

**Authors:** 


### ON THE TREATMENT OF ABSCESS IN THE LIVER. By *Mr. Annesley.*

[Art. IV.]

IN a former number, we introduced Mr. Annesley's observations on the symptomatology of hepatic abscess. He has dedicated a section to the TREATMENT, which we are now to notice.

It is to be borne in mind that, although matter may be actually forming in the liver, yet the inflammatory action which produced it does not cease altogether with that event. In some cases it continues with considerable activity until the abscess either makes its way externally or communicates with some internal viscus. In other instances it greatly subsides, exhibiting only symptoms of constitutional irritation, or hepatic fever. When the formation of abscess is unequivocal, we must endeavour to control all excess of local inflammation which might tend to enlarge the sphere of mischief, taking care, at the same time, to support the general strength for the struggle which must inevitably ensue.

"When, therefore, the general tumefaction and throbbing in the hepatic region, which accompany the early formation of abscess, are considerable, and attended with any degree of pain, firmness of pulse, and excitement of the tongue, local depletions should be instituted, and repeated to an extent which the particular circumstances of the case will point out. When these symptoms are present, and the patient has not had rigors, or cold sweats, or formication, or fainting sensations, or a sense of sinking, with anxiety at the scrobiculus cordis, or night perspirations, then we have not sufficient reason to infer the actual existence of abscess, but it may be imminently impending. In this case our practice must be most decided, and should consist chiefly of large local depletions, which must be repeated until the symptoms of coming danger vanish, or as far as the powers of the system may admit. In those cases where the formation of matter is evident, and the fulness about the margin of the ribs or its vicinity is considerable, the treatment must necessarily depend upon what has been previously done. In these the employment of mercury ought to be entirely laid aside, excepting as a purgative; for attempts to affect the salivary glands with it will generally fail, will merely add irritation to an already irritable pulse, and materially injure the powers of the system—those very powers on which the future recovery of the patient most materially depends. If the local symptoms, and the state of the pulse and of the system, seem to require it, the application of a few leeches in the vicinity of the tumefaction will be generally serviceable; and afterwards poultices should be assiduously employed, with a view of promoting the external pointing of the abscess.

"Although the continuance of mercurial preparations, after the existence of abscess is apparent, with any other view than that of carrying off the morbid biliary and intestinal secretions, (when calomel, in the manner in which we have particularly recommended it, is most suitable,) is improper, yet the nitro-muriatic solution may be employed in any of the modes we have described, or the nitric acid may be used in a state of very weak solution as the common drink. We have never seen any very marked advantage derived from the nitric acid pushed to its utmost extent in abscess of the liver; but in the form of a common drink we consider it beneficial, as being gently tonic and refrigerant, and particularly grateful to the patients, especially when it is found not to disorder the bowels, or add to whatever derangement may be existing in them at the time. It may be also used in combination with tonic infusions." 647.

As the abscess advances externally, the tumefaction is changed to a more distinct tumour, generally softest at its apex, with an extended and hardened base. But if the abscess be seated in the concave surface of the organ, a distinct tumour is seldom to be felt, but only a general tumefaction in the hepatic region. When we have reason to believe that the abscess is pointing upon any internal organ, as the lungs, the stomach, &c. we can do little more than palliate symptoms as they arise, and wait the event. The following are Mr. Annesley's directions for the formidable accident which sometimes happens in these cases.

"If, in consequence of adhesions formed between the walls of the abscess and the diaphragm, and between this latter and the lungs, the abscess empty itself into the bronchia, little else can be done than to palliate the thoracic symptoms attendant upon the disease, and support the strength of the patient. In cases of the above description, we have generally derived much advantage from the exhibition of conium and blue-pill, in the proportion of about two parts of the former to one of the latter; and when the purulent collection has found its way into the lungs, we have generally derived advantage from the nitric acid in combination with laudanum, hyosciamus, or conium. When the tongue remains moist, the expectoration copious, easy, and purulent, and the patient complains of little or no pain, and the pulse is devoid of hardness or sharpness, we have also given the decoction of cinchona, with the acid and the narcotics. During the time that this practice is continued, an aperient draught must be given, with the view of keeping up a gentle action upon the bowels. For this purpose, the bitter aperient mixture, frequently mentioned in the course of the Volume, may be given, either at night or early in the morning. If the patient's strength begins to fail, and if there be night perspirations, and loss of that degree of appetite requisite to support the powers of the system, the tonic decoction, with the acid, as now recommended, should not be omitted; and if, in addition to these symptoms, there be also present signs of general exhaustion, with a weak pulse, a cold, clammy state of the extremities, and cold perspirations, or even a state of the system approaching to this, the decoction of bark should be combined with the spiritus ammoniæ aromaticus, and other warm antispasmodics, in the place of the acid. In cases of this description, particularly when the expectoration is considerable, and no acute or painful symptoms present, the mistura ferri composita may be given and, in order to keep the bowels open at the same time, may be combined with the tinctura aloës composita; and the aloes and myrrh pill may be taken every night at bed-time, in a dose sufficient to procure a full evacuation in the morning." 649.

When the hepatic abscess is pointing on the stomach, as indicated by some difficulty of swallowing, vomiting after food, thirst, a sense of "pumping of the stomach," and other symptoms, palliative medicine is still our only resource.

"With this view we have usually given, when the irritability of the stomach was most urgent, a full dose of calomel with two grains of opium at bed-time, an infusion of calumba with tincture of opium, or the camphorated tincture of opium, and sometimes acids with opiates and antispasmodics; preserving, during the while, a free state of the alvine evacuations, by means of enemata suited to whatever state the bowels may be in at the time." 649.

Bowel-complaint, occasioned mostly from morbid secretions, is a very troublesome attendant on hepatic abscess. Evacuations are necessary; but they should be either conjoined with, or succeeded by, anodynes.

"As soon as the abscess has advanced to that state, in process of pointing externally, which shall offer a fair prospect of advantage from giving an artificial exit to the collected matter, the operation for this purpose should not be delayed. But it ought not to be undertaken precipitately, and before the purulent formation has made its way sufficiently near to the external surface of the organ, or before the part at which it points has formed adhesions to the opposite part of the abdominal paries. The practitioner should also be fully convinced, from the state of the tumour in the hepatic region, and from the history of the case, that abscess actually exists, and that the tumour does not proceed from an excessive accumulation of bile in the gall-bladder." 651.

Obstructions in the ductus communis and other causes sometimes determine great accumulations of viscid bile in the gall-bladder, and thus occasion a tumour at the margin of the false ribs, which has been more than once mistaken for hepatic abscess—and death produced by an operation.

"The accumulation of bile in the gall-bladder, when supervening to congestions of the liver, is often followed, as the formation of matter frequently is, with a remission of the more acute symptoms, and with slight chills: hence the one is more readily mistaken for the other. In the case of abscess, however, the diffused tumefaction preceding the formation of a distinct tumour, with pulsating pain in the region of the liver; the sensation of sinking, with anxiety and oppression; the night sweats, and clammy state of the surface; and the frequent formications or rigors, are generally, of themselves, distinctive of the suppurative process. In the case of great accumulation of bile in the gall-bladder, the tumour is circumscribed, not preceded by a diffused tumefaction, and is equally soft at its base as at its apex. On the contrary, the tumour proceeding from abscess is at first large and diffused, becoming more circumscribed in its progress, and presenting softness or fluctuation at its apex only, whilst the base is harder and more elevated. These points being duly considered, the existence or the non-existence of abscess may be satisfactorily determined." 652.

*External pointing and Operation.* When the abscess proceeds externally, the pain, fulness, and distention in the right hypochondrium and epigastrium, complained of before the suppurative process commenced, increase for a time, and afterwards diminish considerably, leaving, in the place of the diffused fulness and soreness, a tumour, which becomes more and more circumscribed.

"When the abscess advances beneath the false ribs, or near the epigastric region, it is generally sufficiently perceptible; but when it points higher up, or more posteriorly, so as to come beneath the ribs, then a bulging out of the hypochondrium is merely remarked, with fulness of the intercostal spaces, and pain and soreness limited almost entirely to one small spot. In the great majority of abscesses, the direction is to the superior and exterior surface of the liver, and hence their communication so frequently with the diaphragm and lungs, when they fail of pointing more externally. But even in such cases, adhesions to the peritoneum opposite to the seat of abscess are not always formed; for the abscess may advance to the very serous surface of the viscus without coagulable lymph being effused upon this surface in a degree sufficient to attach it to the adjoining parietes of the abdomen. When this is the case, although a circumscribed tumour may point outwardly, yet there will seldom be much redness of its external surface,—an appearance which always indicates that adhesions have formed, or are far advanced in the process of formation; and when this is observed, with diminution of the surrounding fulness which accompanied the formation of abscess, and with fluctuation of matter in the tumour, then the operation may be undertaken with every prospect of success. But it ought never to be undertaken too early, or before these signs of maturity are present, for reasons which we shall soon have occasion fully to state." 653.

In many cases nearly the whole of the right lobe becomes one enormous abscess—"yet there may not be much destruction of the internal structure of the organ, it being impacted around the parietes of the purulent collection." In other cases, however, the parenchymatous structure of the liver is broken down and destroyed—and here, of course, an operation can be of little use.

"When the liver is considerably enlarged in the early stage of inflammation of its internal texture, the formation of abscess is often with difficulty prevented; and when it is felt below the ribs, with general fulness over the hypochondriac and epigastric regions, the constitutional symptoms already detailed being also present, we may consider that suppuration is going forward, although abscess has not actually arrived at its height. But as long as there are tension, hardness, and tumefaction in the region of the liver, we may be assured that the suppurative process is not yet complete. As soon, however, as the abscess is completely formed, the pain and general tumefaction are diminished; the liver, in some cases, seems to shrink into its natural position, leaving merely a more or less distinct tumour near the centre of the general tumefaction, when the abscess points externally, unless the purulent collection be so extensive as to fill the greater part of the abdomen." 653.

So long, therefore, as there are pain and tenderness felt in the region of the liver, with an undefined tumefaction, and neither a circumscribed nor soft fluctuating tumour, an operation would be premature.

"But when the pain and general fulness are diminished and replaced by a distinct tumour, without acute pain, soft and fluctuating at its apex, or with a soft elasticity and slight lividity or redness of the surface, and a somewhat hardened and elevated base, indicating that the purulent matter has found its way to the external covering of the liver at the tumid part, and that the liver has there formed adhesions to the opposite part of the abdominal parietes,—the operation may be undertaken with every expectation of success. If it were performed during the former condition of disease, the operator would have to cut deep into the substance of the organ before the purulent collection could be reached, and would run the risk of almost immediately destroying the patient from the hæmorrhage consequent upon cutting deep into so vascular an organ as the liver is, with the additional hazard of finding no adhesions formed between the side and the seat of disease. This is a point of great importance in practice, and should be most particularly attended to, as a mistake of the kind now alluded to would prove fatal." 654.

Mr. Annesley does not recommend the operation to be performed by means of the trocar, as is usually done—and for the following reasons:—

"The pus which is formed in abscess of the liver is often full of large flakes, and sometimes contains large coagulated clots of a cheese or curd-like matter, which will not pass through the largest trocar, the more fluid portions only coming away. These clots remain, acting as foreign substances in promoting continued suppuration of the organ, and febrile excitement of the system. We, therefore, have been always in the habit of performing the operation without the trocar, and in the following manner:—having made the external incision large, and with caution, until the peritoneum is fully exposed, the fluctuation of the abscess will be distinctly felt. An abscess-lancet should then be introduced, and the tumour laid open to the full extent of the external wound, which ought to be from two and a half to three inches in length. Care should always be taken that the opening extend not beyond the limits of the adhesions which have been formed. The purulent collection being fully evacuated, the cavity should be filled with lint, which gives a mechanical support to the excavated parts, and the wound dressed with compresses and bandages in the usual way." 655.

The wound must be dressed twice a day, after the operation, for a short time—the bowels gently acted on, and light nutritious food exhibited. Tonics, and even wine will be necessary, if the powers of life seem to flag.—The two following cases will sufficiently illustrate the successful termination of hepatic abscess by operation.

"*Case* 1. Captain N——of the Madras army, was attacked early in December, 1817, in camp on the Narbuddah, with an acute inflammation in the liver, for which he was very boldly and decidedly treated. The army were actively employed at this time in Malwah, and we had not many opportunities of visiting him; but we did occasionally see him, and fully concurred in the views and mode of treatment adopted by his medical adviser at the time, Mr. H. Jones, a very able practitioner. In defiance, however, of every effort to prevent the formation of matter, a very large abscess formed, and pointed a little below the margin of the ribs, on the right side. Early in February the abscess became sufficiently prominent, with evident fluctuation and adhesions to the parietes of the abdomen, and an operation was determined upon, in which we were assisted by Mr. Jones. An incision was made, two inches and a half or three inches in length, through the most depending part of the tumour, and carried carefully down to the peritoneum, which was also dissected through till the abscess was distinctly visible, when an abscess-lancet was introduced, and the tumour was laid open to the full extent of the external wound. About a quart of pus was discharged, full of those flaky, ropy clots of pus which we have frequently found on examining similar cases of the disease in the dead subject. The empty space was completely filled with lint, the wound dressed with compresses and bandages, and there was much less exhaustion than we had often seen before, when much less discharge had come away. The dressings were removed morning and evening. The discharge was free and copious. He had no fever after the operation, but improved daily; and although the weather was very hot, and the army marching daily through jungly and unwholesome countries, namely the Narbuddah and Sindwah jungles, and Candeish, there was none of those bad symptoms after the operation, which we have invariably seen when the trocar was used, and he progressively recovered. He did not, however, perfectly regain his strength till he took a sea-voyage, and remained a few months at the Cape of Good Hope, after which he returned to his duty in India, and is now a healthy man. The treatment, after the operation, consisted of gentle tonics and aperients, directed with the view of supporting his strength and restoring the healthy functions of the abdominal viscera." 657.

*Case* 2. "An European woman, about 25 years of age, wife of a soldier in His Majesty's 46th Regiment of Foot, came into the General Hospital at Madras, in August, 1819, with a very large tumour in the right side, extending considerably below the ribs, and across the epigastric region to the left side. There could be no doubt of the liver being the seat of disease; but abscess, though evidently forming, had not arrived at its maturity. The region of the liver was hard and painful to the touch, and there were considerable tumefaction and heat of skin; irritative fever; foul tongue; full, quick pulse; bowels constipated, and considerable dyspnœa and oppression at the præcordia. Fomentations and poulticing, aperients, saline mixture, &c. &c. were used for ten or twelve days, when the tumefaction subsided very considerably, and an evident fluctuation was discovered below the præcordia, and near the margin of the ribs, which appeared to point in this direction, but not sufficiently to authorise an operation. These appearances were encouraged by the same remedies for some days longer; and although it did not point so distinctly as in Captain N———'s case, yet, as the abscess was evidently matured, of which we judged from the subsiding of the general tumefaction, and the evident tumour and fluctuation, which, although obscure, from its being deeply situated, could not well be mistaken. We determined, therefore, on the operation, and, in presence of the surgeon of the regiment, assistant-surgeons, and some medical men of the Presidency, we made an incision through the integuments over the centre of the soft tumour, which we found exceedingly thick, with much adipous matter underneath, and which accounted for the obscure feel of fluctuation. From the external surface to the peritoneum the wound was at least three inches and a half deep. On laying the peritoneum bare, we found the abscess distinctly pointing: an abscess-lancet was introduced, and the opening enlarged to the full extent of the external wound; nearly two quarts of green, watery fluid came away, with thick, flaky, and ropy matter. The cavity was filled with lint, and dressed in the usual way, with a large poultice over the whole. She was much relieved from oppression; her spirits were good; and in the evening she was as well as could be expected. The discharge was very copious, and consisted of coloured matter similar to what was removed by the operation. An anodyne was given at bed-time. The wound was dressed in the same manner, that is, filling the cavity with lint; and in two or three days the pus became more healthy, of a white colour, but still ropy and flaky. In a week the discharge diminished much, and considerably less lint was required for dressing; and in sixteen days the dressings were simple, the discharge trifling, and the strength improving. In six weeks she was perfectly well, and enjoyed good health while she remained in the garrison. The regiment to which her husband belonged left Madras about eight months afterwards, and she accompanied him, in perfect health." 658.

*Case* 3.—Abscess opening into the Lungs. This case we shall abbreviate. Captain E———, of the Madras army, arrived at that Presidency on the 15th of March. He had been ill since January with liver affection, but not finding relief at Masulepatam, he came round by sea to Madras. The vessel being detained

27 days on the passage, the patient suffered much inconvenience, and, on the night before his landing, threw up a quantity of purulent matter by coughing. He complained of great weight in his side, and there was visible enlargement of the liver. It was evident that an abscess had burst into the lungs. There was no external pointing to justify an operation. His strength was supported by nutritious food—a large poultice was applied to the abdomen, and the nitro-muriatic lotion to the side. This plan was pursued for ten days, and with apparent advantage. His strength improved, and the external swelling decreased; but he felt more internal fulness and pain than previously, particularly between the tenth and eleventh ribs, where there was an obscure fluctuation. Mr. Richardson thought an operation advisable; but, in a consultation, this was negatived. But the patient insisted on the operation, and accordingly an incision was made between the tenth and eleventh ribs.

"The wound was made about three inches in extent; and, on introducing my finger, I found the abscess considerably higher than where we had made the opening, although this appeared to be the most depending part of the tumour. From the wound being large, we were enabled to pass two or three fingers, so as to support the tumour, and with the point of a scalpel we opened it, making our hand the medium of conducting the pus, for we found the abscess pendulous and the adhesions doubtful. A considerable quantity of curdled pus was discharged, to the amount of at least sixteen ounces. The wound was afterwards cautiously filled with lint and dressed, and a large poultice was applied over the wound and hypochondriac region. The patient passed a much better night than usual, and the following morning the matter flowed freely from the wound, and was thick, flaky, and curdled. His bowels were very costive, but were attended to, and relieved daily. The pulse was quick, but of good volume; appetite tolerably good. From this time he improved; his sleep became more quiet; cough less troublesome; bloody sputa ceased, though the expectoration continued purulent. No change whatever was made in the treatment; he was dressed twice a day. The discharge from the wound diminished; granulations formed; the pulse was frequent, 100 in a minute, but of good strength; tongue became healthy, clean, and natural; and, in order to avoid the hot season, he embarked on the 6th April, on board the David Scott, for the Cape of Good Hope, with every prospect of recovery. We have since learned that he had a relapse on board ship, and died, either at sea or at the Cape of Good Hope." 660.

The credit of the above operation is justly given to Mr. Richardson, whose science and judgment Mr. A. had often occasion to admire. This excellent medical officer has since fallen a victim to cholera.

Two cases are next related, where abscess of the liver had burst into the lungs, and yet recovery took place—rare instances, indeed, of good fortune. One of these cases is so remarkable, that we are tempted to extract it.

Case 4.—Abscess of the Liver breaking into the Lungs.—Recovery.

"Owen Keating, ætat. 29. His Majesty's 53d Regiment. 3d July, 1819. Admitted this morning into the Madras General Hospital, with dysenteric symptoms; complains also of pains in the right hypochondriac region; stools of a green colour, scanty, no tenesmus; pulse and skin natural; tongue foul; very thirsty.—Sumat. hydr. submur. gr. x. stat. post horas tres; ol. ricin. ℥j.

"*Vespere.*—Bowels freely opened.—Repet. calom. gr. x. cum pulv. antim. gr. iij. h. s. Capiat haust. salin. tertiâ quâque horâ.

"4th.—Feels better this morning; was purged four times in the night; tongue loaded; has pain in his side, under his fourth rib, when he drinks any thing; pulse small.—Mist. purg.

"5th.—Tongue black and loaded; still feels pain in his side; pulse hurried and quick; face pale; stools clay colour; has taken the saline mixture.—Pilul. hydr. cum calom. three times a day. Haust. amar. cum sennâ, ℥ij. nocte maneque.

"6th.—Feels much better; stools green colour, with hardened fæces; tongue black; pulse small and hurried; was only purged twice.—Mist. purg. ℥iij. stat. Cont. ut anteà.

"7th.—Tongue continues black and furred; stools yellow and watery; pulse small; skin cold.—Enema stat. ℞ Aquæ ammon. ♏xxx.; spirit. laven. comp. ʒj.; aq. menth. ℥ij. M. ft. haust. stat. Repet. pilul. et haust. amar. cum sennâ, ut anteà.

"No change in the symptoms occurred till the 13th.—The pills, enema, and bitter aperient draughts, were continued.

"13th.—Tongue lost the blackness entirely; he is better in every respect—Cont.

"*Three o'Clock*, p.m.—Seized with violent pain in the region of the liver, which impedes his breathing, attended with cough, and a sudden and great expectoration of pus; skin rather cold, with a profuse perspiration over it; pulse feeble.—℞ Æth. vit. ʒj.; mist. camph. ℥jss. M. ft. haust. stat. cap. Fotus parti dolenti.

"*Six o'Clock*, p.m.—Pulse 122 and small; skin warm, with perspiration; tongue loaded with a yellow mucus; cough continues, with copious expectoration; about three pounds altogether have been discharged; pain particularly felt just under the margin of the ribs of the right side and up towards the breast; breathing still considerably impeded.—Cont. haust. cum æth. sulph. et mist. camph. ut anteà. Repet. fotus.

"*Half-past Six o'Clock*, p.m.—Pain continues very acute about his right breast. —App. hirud. jv. parti dolenti. Cont. fomentation and draught.

"14th.—Pulse 114, small and fluttering; skin cool and with a cold moisture; a rough noise in his chest when he breathes; as if the bronchi were filled with matter; eyes lively; pupils less dilated than they were; expectoration of matter ceased last night at nine o'clock; still feels pain in the right side between the diaphragm and the superior convex surface of the liver; has very little cough, but he is evidently oppressed; has had stools this morning and walked to the commode.—Keep his bowels open with enemata. He feels relief from the draughts. Cont. them and the fomentations. Arrow-root, milk, &c.

"*Evening*.—Sputa pure pus; pulse 140 in a minute; skin warm and moist; less expectoration; breathing easier; his belly open; tongue furred and yellow, not dry. —Mist. salin. febrif. cum antim. tart. gr. jss.

"15th.—Difficulty of breathing excessive; there seems fulness over the whole abdomen: pulse 120; skin moist and warm; has not had a stool since yesterday morning.—Enema. Cont. foment. Repet. mist.; and apply a warm plaster over the chest.

"*Vespere*.—Pulse 116, very weak; skin warm and covered with moisture; less pain, and he breathes better; his bowels have been well opened; stools perfectly good.—Cont.

"16th.—Pulse 112, more firm than it was; skin more natural; tongue rather foul, not black; feels easier; some oppression still in his breathing, but he has less pain generally; sputa pure pus, but not so much in quantity as before; bowels regular.—Repet. enema. Cont. ut anteà.

"*Vespere*.—Continues nearly the same; bowels opened by the enema; expectorates occasionally.—Cont. mist. salin.

"*Nocte*.—Cough rather troublesome.—℞ Tinct. opii camph. ʒij.; æth. vit. ʒj.; stat. Mist. camph. ℥jss.

"17th.—Pulse 116, small, and rather weak; skin cool, and a moisture over his forehead; tongue yellow; pain in his breast easier, and he breathes with more freedom; sputa thick tenacious mucus, with spots of pus; bowels very well.—Cont.

"*Evening*.—A wound which he received in his thigh has broke out and discharged a quantity of pus, with several small stones; pulse 112, very irregular and fluttering; skin of his hands cold, and covered with perspiration, perhaps from exposure to the air, as his body feels warm; he has much less pain in his chest, and breathes with more freedom; the wound in his thigh was fomented, and a quantity of pus came away, but no stones.—℞ Magnes. sulph. ʒv.; aquæ menth. pip. ℥iij.; tinct. lavend. comp. ʒss. M.; p. m. s. Cont. mist. ut anteà.

"18th.—Pulse very small and weak; skin cold, and rather clammy; says he is much easier; not so free an expectoration; there was a considerable discharge from the wound in his thigh.—Con. med. Sago and port wine.

"*Vespere.*—Pulse continues quick and small, 120 in a minute; skin warm, but still covered with perspiration; stools copious, feculent, and of a natural colour; tongue rather cleaner; some thirst; cough somewhat troublesome, but very little expectoration; side and breast easy, as also his thigh; took the sago and wine very well to-day for his breakfast and dinner.—℞ Decoct. cinchon. ℥jss.; tinct. cinchon. ʒij.; acid. sulph. dilut. ♏xx. M. ft. haust. tertiâ quâque horâ sumend.

"19th.—Pulse firmer, 110 in a minute; skin more natural; says he is much better; no matter in his expectoration.—Cont. cort. ut anteà.

"20th.—Pulse hurried and confused, 120 in a minute; expectoration difficult; cough troublesome, and some uneasiness in his chest; voice hoarse; skin natural.—Repet. mist. purg. ℞ Mist. camph. ℥jss.; tinct. opii. comp. ʒj.; tinct. scillæ, ♏xx.; sacch. alb. q. s. Ft. haust. stat. sumend. Cont. haust. cinchon. ut anteà.

"21st.—Pulse 108, not strong; feels easy; very little expectoration; oppression less; passed a good night, but seems to breathe quick; skin natural.—Cont.

"22d.—Pulse small and fluttering, 99; is stuffed in his chest, and his countenance is bloated; no pain in his breast, or difficulty of breathing, but has pain in the region of the liver; he cannot lie at all on the left side, and he has a short cough; bowels regular; stools natural; skin covered with moisture; considerable fulness in the right hypochondriac region, and the appearance of a large tumour, but he has no pain, except along the margin of the ribs to the spine.—Apply a large poultice round the whole hypochondriac and epigastric regions, with unguent. mercur. ʒij. Cont. haust. camph. bis in die. Cont. cort. Per. ut anteà. A flannel shirt.

"23d.—Fulness over the whole abdomen, and a tumour formed about three inches from the spine, between the fifth and sixth ribs; he cannot lie on the left side, but can stand up without any difficulty; stools natural; pulse hurried and weak; 110 in a minute.—Cont. poultice. Cont. med. ut anteà.

"25th.—Looks much better; fulness in the hypochondrium, and general swelled appearance of his face, neck, and chest, gone; breathing still rather oppressed; no pain in his chest; short cough continues; pulse hurried and confused; skin cool and moist; bowels regular; stools feculent and natural; tumour in the side seems to be diminished, but there continues an evident fulness.—Cont. ut anteà.

"27th.—Continues much better; the tumour towards the posterior part of the liver considerably decreased; pulse not so irregular, and firmer; skin natural; bowels regular.—Habeat pot. acid. nitros. ad libitum. Cont. omnia.

"30th.—The oppression in his chest is better; his pulse 96, and firm; feels slight pain in the right side, immediately under the hypochondriac region.—Apply three leeches.—Cont. med. omnia.

"31st.—Feels pretty easy; some tension in the epigastric region still; bowels open; felt relieved after the application of the leeches, complained of yesterday.—Cont. omnia. Nitro-muriatic wash to be applied to the abdomen.

"*August* 1st.—Continues much better; bowels regular; pulse 100, and pretty firm; skin natural; tongue clean; cough less; side quite easy.—℞ Decoct. cinchon. ℥ij.; tinct. cinchon. ʒij.; acid. sulph. dilu. ♏xx. M. ft. haust. quartis horis capiend. Cont. acid. nitros. ad libitum, ut anteâ; nec non nocte maneque, lotio pro abdom. Vin. rub. ℥vj.

"4th.—The oppression in his breast removed, and he is recovering rapidly; cough very slight.—Cont. omnia.

"6th.—Says he feels quite well, although he seems to have some oppression still when he breathes; skin natural; tongue clean; appetite good.—Cont, haust. cinchon. et vin. ut anteà.

"7th.—Perfectly free from pain and uneasiness; looks greatly improved; bowels regular.—Cont.

"From this date he continued to recover, but was not discharged for a considerable time, in order to prevent any relapse before his health was completely restored. He recovered perfectly." 676.

We have now, in four articles, concluded our analysis of the first volume of this immense and highly valuable work; and shall pursue our labours continuously through the remaining volume. The profession, as well as the author, must be well aware that the contents of these volumes would be comparatively lost to the public, were they not thus diffused through the medium of an analytical review. We shall spare no pains in rendering the results of Mr. Annesley's investigations available by his brethren at large.

---

